# Medical and Physical Intelligence

**Published:** 1806-02

**Authors:** 


### MEDICAL AND PHYSICAL INTELLIGENCE.

---

### *Small-Pox and Inoculation Hospitals, St. Pancras.*

At an Half-yearly General Court, held at Batson's Coffee House, Cornhill, London, on Thursday the 19th Day of December, 1805, Jonathan Hoare, Esq. V. P. in the Chair.

Dr. Adams, Physician to these Hospitals, made the following Report.

The great success of Vaccination, and its consequent almost universal adoption, had reduced the Small-pox to that state in which it is described by those authors who practiced before Inoculation was known in England; that is, the disease, after declining for more than seven years, on the eighth became epidemic; according to the language of Sydenham, sparing neither age nor sex, among those who remained liable to it. It is impossible to say, how extensive the mortality might have proved, had not the industry of the friends of vaccination previously induced so many to go through that operation.

The

The number, however, who remained liable to the disease was still considerable, particularly among the poor, who, as Dr. Heberden observes, are too often obliged to be inattentive to the preservation of their Children.

A great proportion of such as applied for the assistance of the hospital, were in a state truly alarming from the hour of their admission; and though the medical officers trust their diligence has been proportionate to the urgency of the times, it would be melancholy to relate in how many instances all the services they could render, were to soften the pains of approaching dissolution.

The names and places of abode of each patient being registered, Mr. Wachsell had an opportunity of learning the state of each neighbourhood respecting the disease, and of urging the mothers to vaccinate their children, till by degrees the general alarm induced many to make the first application, who had hitherto resisted every other consideration.

At first, small-pox inoculation was refused to such as came from neighbourhoods not already infected with the casual disease. But those places became every day fewer, till the disease spread over the whole metropolis, and most of the populous towns in the kingdom.

From this statement, the Court will perceive, that the increased inoculation for small-pox has been the *consequence*, and not the *cause*, of the recent epidemic state of the disease. If any further proof of this were necessary, it might be urged, that whilst inoculation was the most unrestrained, the epidemic has subsided by the unknown means that produced it.

In the course of our attention to every circumstance of the disease, on account of which the liberality of the Governors has expended so much time, and every other consideration, it was discovered that no casual cases had occurred, but in children born since the year 1796, or in adults who had resorted to the metropolis since that period. The females were most of them in service, many of the males of the same description; but still more, private soldiers, or labourers in factories and other large undertakings.

Though the epidemic state of the disease has, at present, greatly subsided, there is too much reason to fear, by the records of what has formerly happened, that it may return with the same violence in the spring.

The COURT, after considering the above Report,

Resolved,—That in order to lessen as much as possible the extent and fatality of the disease, this Court do recommend to all conductors of large manufactories, and other extensive concerns, to ascertain whether any persons in their employment have had the small-pox, or been vaccinated; and to the heads of all families, to renew a custom, once very general, and which it is much to be regretted should ever have been discontinued, of making similar inquiries relative to servants offering themselves for hire.

And

And this Court, whilst they regret with the sincerest concern the extensive fatality of the small-pox for the last ten months, reflect with no less satisfaction on the means, under Divine Providence, which these hospitals have afforded of alleviating this dreadful calamity; and being desirous, more especially whilst the present epidemic continues, that as few difficulties as possible may exist to the reception of casual patients at the Small-pox Hospital, have directed that the most ample instructions shall be given by their physician, their resident apothecary, and their secretary, for facilitating the admission.

As the most effectual assistance can be given in the early stage of the disease, they wish it to be known, that where symptoms sufficiently suspicious occur, attested by any medical practitioner, such patients may be received into the hospital before the eruption appears.

That variolous inoculation of out-patients is confined as usual to children under five years of age; these may, nevertheless, be admitted into the house with their mothers or nurses, who will be required to contribute to the charity towards their own subsistence.

That vaccination will be given to all persons who may apply at the Inoculation Hospital, daily from ten to twelve o'clock in the forenoon; Sundays excepted.

That it appears by the Returns made to this Court, that all the departments of this Institution have been extensively occupied during the current year, and particularly during the last seven months, in which it has given relief to 3,671 patients; this affords an ample testimony of its utility, and to a continued attention to the principles of its foundation, which were, not only to relieve the actual sufferer under the danger and distress of so fatal a disease, but also to preserve the indigent as far as possible from the terrors of its invasion.

That for these beneficent purposes, the Committee of this Charity were amongst the first enquirers into the advantages of vaccination; and it is with grateful pleasure they have been enabled to state, that the most numerous, decisive, and satisfactory trials of that invaluable discovery were first made at the Inoculation Hospital: their constant success has confirmed all that was promised by it, and their vast accumulation of evidence gave the earliest confidence to the public of its permanent security.

And although this confidence may have been affected by some cases, by no means more numerous than ought to have been expected; yet that it appears by the register, that the numbers vaccinated at the hospital within the last twelve months have nearly equalled those who have been inoculated; and that the aggregate has been considerably greater than in any former period of the same extent.

By the Court,

*No. 40, Haydon-Square, Minories.* A. HIGHMORE, Secretary.

*An*

*An Account of* PATIENTS *received into the Small-Pox and Inoculation Hospitals, and of* OUT-PATIENTS, *in* 1805.

| | IN-PATIENTS. | | | OUT-PATIENTS. | | | |
|---|---|---|---|---|---|---|---|
| | *Nat. Small-pox.* | *Vaccinated.* | *Inoculated.* | *Natural Small-Pox.* | *Vaccinated.* | *Inoculated.* | *Total.* |
| January | 5 | 3 | 12 | | 32 | 18 | |
| February | 2 | 1 | 15 | | 50 | 34 | |
| March | 5 | 5 | 18 | | 120 | 166 | |
| April | 5 | 1 | 28 | During | 316 | 338 | |
| May | 17 | 6 | 30 | the Year, | 450 | 210 | |
| June | 26 | 5 | 11 | | 273 | 74 | |
| July | 31 | 8 | 15 | 35 | 216 | 134 | |
| August | 39 | 7 | 28 | | 165 | 153 | |
| September | 25 | 4 | 23 | | 195 | 418 | |
| October | 46 | 4 | 54 | | 157 | 497 | |
| November | 48 | 3 | 33 | | 46 | 230 | |
| December | 31 | 3 | 33 | | 26 | 66 | |
| | 280 | 50 | 300 | 35 | 2,046 | 2,338 | 5,049 |
| Natural Small-pox Patients, previous to 1805 - | | | | | | | 21,083 |
| Inoculated Patients, ditto - - - - - | | | | | | | 36,378 |
| Vaccinated, ditto - - - - - - | | | | | | | 17,261 |
| | | | | | | | 79,771 |

JOHN CHRISTIAN WACHSELL,

*Jan.* 2, 1806. Apothecary and Steward.

---

Mr. BISHOP, in a letter to the Editors, dated Kirton, near Boston, Lincolnshire, Jan. 14, 1806, says,—"I would strongly recommend to your readers and other medical men, the following simple method of treating leeches, and am convinced, from many years experience, of the great advantage that will accrue from it, particularly as they are become so very scarce and dear: Immediately on leeches falling from a part to which they have been applied, I immerse them in water as warm as new milk, where they are to remain an hour; they are afterwards to be treated in the common way, observing to change the water every second day; they should not be returned to the old stock for at least a week. No means should be made use of to induce the leech to eject the blood it has imbibed. I am preparing a place for the purpose of breeding leeches; should I be successful, I will inform you."

---

The Spring Course of Lectures at St. THOMAS's and GUY's Hospitals, will commence in the following order.—At St. Thomas's:

Thomas's: Anatomy and the Operations of Surgery, by Mr. CLINE and Mr. ASTLEY COOPER, the 21st of February. The Principles and Practice of Surgery, by Mr. ASTLEY COOPER.—At Guy's: Practice of Medicine, by Dr. BABINGTON and Dr. CURRY, the beginning of February. Chemistry by Dr. BABINGTON and Mr. ALLEN. Theory of Medicine and Materia Medica, by Dr. CURRY. Experimental Philosophy, by Mr. ALLEN. Midwifery, and the Diseases of Women and Children, by Dr. HAIGHTON. Phisiology, or Laws of the Animal Œconomy, by Dr. HAIGHTON. Clinical Lectures on select Medical Cases, by Dr. BABINGTON, Dr. CURRY, and Dr. MARCET. On the Structure and Diseases of the Teeth, by Mr. FOX. On Veterinary Medicine, by Mr. COLEMAN. These several Lectures are so arranged, that no two of them interfere with each other in the hours of attendance; and the whole is calculated to form a complete Course of Medical and Surgical Instruction. Terms and other particulars to be learnt of Mr. STOCKER, apothecary to Guy's Hospital; who is also empowered to enter gentlemen as pupils to such of the Lectures as are given at Guy's.

---

Mr. MILBURNE's Spring Course of Lectures on Anatomy, Phisiology, and Operations of Surgery, will commence on Monday the 24th of February, at his house in St. James's-street.

---

Mr. MOOR, Surgeon Dentist to Her Royal Highness the Duchess of York, will commence a Course of Spring Lectures on the Structure and Diseases of the Teeth on the 10th of February, wherein will be shewn the various Operations, and the entire Practice of the Dentist explained. Further particulars may be known by applying at his house, No. 6, Palsgrave Place, Temple.

---

Mr. J. C. SAUNDERS, Demonstrator of Practical Anatomy in the Anatomical School of St. Thomas's Hospital, and Surgeon to the London Dispensary for Diseases of the Eye and Ear, has for a considerable time been preparing for publication An Illustration of the Anatomy of the Human Ear, accompanied by Views of that Organ, accurately drawn, of the natural Size, from a Series of Dissections. To which he intends to add a Treatise on its Diseases, the Causes of Deafness, and its proper Treatment. The work will be published on the first of March.

---

Dr. HARRISON intends shortly to publish a pamphlet on the Imperfect State of the Practice of Physic in Great Britain; to which will be added, Hints for its Improvement, &c.

---

Mr. JOHN HUNT, Author of Historical Surgery, &c. proposes to publish by subscription, Anatomical Speculations on the Form of Animals, and on the new Opinions of Henry Cline, Esq. Surgeon.

Dr.

Dr. Henderson has in preparation for the preſs a tranſlation, with additional Notes, of M. Cabanis's valuable work, entitled, Coup d'Œil ſur les Révolutions et ſur la Réforme de la Médecine, of which we have already given ſome account in our Journal.

---

### *Diseases and Casualties in London in the Year* 1805.

( From the Bill of Mortality. )

| | | | | | |
|---|---|---|---|---|---|
| Abortive and Stillborn | 716 | Grief | 2 | St. Vitus's Dance | 1 |
| Abſceſs | 86 | Headmouldſhot, Horſeſhoehead, and Water in the Head | 157 | Surfeit | 2 |
| Aged | 1452 | Jaundice | 64 | Teeth | 507 |
| Ague | 3 | Jaw Locked | 2 | Thruſh | 108 |
| Apoplexy & Suddenly | 421 | Impoſthume | 1 | Tumour | 2 |
| Aſthma and Phthiſic | 471 | Inflammation | 570 | Vomiting and Looſeneſs | 2 |
| Bedridden | 3 | Influenza | 2 | Worms | 12 |
| Bile | 1 | Inoculation | 1 | Broken Limbs | 3 |
| Bleeding | 23 | Itch | 1 | Broken Neck | 2 |
| Burſten and Rupture | 16 | Lethargy | 1 | Bruiſed | 3 |
| Cancer | 59 | Livergrown | 10 | Burnt | 23 |
| Chicken Pox | 1 | Lumbago | 1 | Choaked | 1 |
| Chin Cough | 1 | Lunatick | 158 | Drowned | 115 |
| Childbed | 222 | Meaſles | 523 | Exceſſive Drinking | 4 |
| Colds | 8 | Miſcarriage | 3 | Executed | 6 |
| Colic, Gripes, &c. | 12 | Mortification | 318 | Found Dead | 3 |
| Conſumption | 3432 | Palſy | 136 | Fractured | 3 |
| Convulſions | 3053 | Palpitation of the Heart | 7 | Frozen | 1 |
| Cough, and Hooping Cough | 703 | Piles | 2 | Killed by Falls and ſeveral other Accidents | 56 |
| Cow Pox | 1 | Pleuriſy | 24 | Killed by Fighting | 1 |
| Cramp | 3 | Quinſy | 4 | Killed Themſelves | 19 |
| Croup | 29 | Raſh | 1 | Murdered | 4 |
| Diabetes | 1 | Rheumatiſm | 10 | Overlaid | 1 |
| Dropſy | 712 | Scurvy | 2 | Poiſoned | 2 |
| Eaten by Lice | 1 | Small Pox | 1685 | Scalded | 10 |
| Evil | 7 | Sore Throat | 8 | Shot | 2 |
| Fevers of all kinds | 1307 | Sores and Ulcers | 6 | Smothered | 1 |
| Fiſtula | 3 | St. Anthony's Fire | 2 | Starved | 1 |
| Flux | 4 | Spaſm | 11 | Strangled | 1 |
| French Pox | 49 | Stoppage in the Stom. | 14 | Suffocated | 2 |
| Gout | 124 | | | | |
| Gravel, Stone, and Stranguary | 17 | | | | |

| | | | |
|---|---|---|---|
| Chriſtened | Males | 10513 | In all 20295 |
| | Females | 9782 | |
| Buried | Males | 8874 | In all 17565 |
| | Females | 8691 | |

Whereof have died,

| | | | |
|---|---|---|---|
| Under Two Years of Age | 5204 | Fifty and Sixty | 1504 |
| Between Two and Five | 2199 | Sixty and Seventy | 1187 |
| Five and Ten | 826 | Seventy and Eighty | 757 |
| Ten and Twenty | 934 | Eighty and Ninety | 390 |
| Twenty and Thirty | 1283 | Ninety and a Hundred | 82 |
| Thirty and Forty | 1765 | A Hundred | 5 |
| Forty and Fifty | 1829 | | |

Increaſed in the Burials this Year 527.

